# Physicochemical Properties of Collagen from *Acaudina Molpadioides* and Its Protective Effects against H_2_O_2_-Induced Injury in RAW264.7 Cells

**DOI:** 10.3390/md18070370

**Published:** 2020-07-18

**Authors:** Jie Li, Yan Li, Yuyao Li, Zuisu Yang, Huoxi Jin

**Affiliations:** Zhejiang Provincial Engineering Technology Research Center of Marine Biomedical Products, School of Food and Pharmacy, Zhejiang Ocean University, 1 Haida South Road, Dinghai District, Zhoushan 316022, China; lijie1749@126.com (J.L.); m18625623399@163.com (Y.L.); lyy699902@163.com (Y.L.); abc1967@126.com (Z.Y.)

**Keywords:** *Acaudina molpadioides*, collagen, physicochemical property, oxidative damage

## Abstract

Collagen is a promising biomaterial used in the beauty and biomedical industries. In this study, the physicochemical characterization, antioxidant activities, and protective effects against H_2_O_2_-induced injury of collagen isolated from *Acaudina molpadioides* were investigated. The amino acid composition analysis showed that the collagen was rich in glycine (Gly), alanine (Ala), and glutamic acid (Glu), but poor in tyrosine (Tyr) and phenylalanine (Phe). Zeta potential analysis revealed that the isoelectric point (pI) of collagen from *Acaudina molpadioides* was about 4.25. It possessed moderate scavenging activities of 2,2-diphenyl-1-picrylhydrazyl (DPPH) and 2,2’-azino-bis-3-ethylbenzothiazoline-6-sulfonic acid (ABTS) radicals in a dose-dependent manner. In addition, the collagen was able to effectively improve cell viability and morphology, inhibit the production of Malondialdehyde (MDA), and increase the activities of Superoxide Dismutase (SOD) and Glutathione Peroxidase (GSH-Px) in cultured RAW264.7 cells, resulting in a protective effect against H_2_O_2_-induced injury. Overall, the results showed that collagen extracted from *A. molpadioides* has promising prospects in the beauty and cosmetics industries.

## 1. Introduction

Collagen is a major structural protein that is widely found in multicellular organisms and constitutes about 30% of the total protein in many cases [1,2]. Collagen possesses a set of specific mechanical and biochemical properties, such as a weak solubility in water, high tensile strength, and the ability to bind water and form aqueous gels, which have resulted in its broad applications in the food industry [3,4]. In addition, due to its low antigenicity, good biocompatibility, and its biological activity, collagen could be used in the fields of medicine and healthcare, in applications such as burn and trauma treatment, beauty, tissue repair, and wound hemostasis. Over the past decade, the enzymatic hydrolysis of collagen for the production of functional peptides with antimicrobial, antioxidant, and antitumor activities has emerged as a promising new field [5,6]. Therefore, the demand of collagen from animal sources has been increasing year by year, due to its emerging and widespread applications.

Currently, at least 29 types of collagen have been identified and characterized from animal skin, bone, swim bladder, and cartilage tissues [7,8]. Each type of collagen from a different tissue has its own unique amino acid composition, molecular weight, and sequence, which help determine the differences in quality, biophysical properties, and peptide bioactivity found across different collagens [2]. It has been shown that the environmental temperatures in which different animals reside are highly related to the amino acid compositions and thermal stabilities of their collagens. The thermal stability of collagens from cold water animals, for example, are generally relatively poorer than those from warm water and land animals [9]. The ocean is rich in biological resources; thus, potential sources of collagen derived from marine animals have attracted increasing attention in recent years. Yu et al. extracted and characterized collagens from the spines and skulls of skipjack tuna (*Katsuwonus pelamis*), and found type I collagens with denaturation temperatures of only about 17 °C [10]. The collagen from the skin of the giant croaker was shown to be a type I collagen and had a decrease in solubility in the presence of sodium chloride [11]. Zhao et al. found that collagen from the swim bladders of miiuy croakers showed 26.7 °C of the denaturation temperatures and antioxidant functions, with potential cosmeceutical applications [8].

The sea cucumber is an important marine resource, with over 1400 species around the world, many of which are edible and have high medicinal value [12]. The body wall of the sea cucumber is rich in protein, of which collagen accounts for 70%. Thus far, the preparation and characterization of collagen from *Parastichopus californicus* [13], *Stichopus vastus* [14], *Holothuria parva* [15], *Stichopus japonicus* [16], *Stichopus monotuberculatus* [17], and *Acaudina leucoprocta* [18] have been reported. Furthermore, bioactive peptides produced by the hydrolysis of sea cucumber collagen have also been extensively studied [19,20,21]. One highly valued sea cucumber, *Acaudina molpadioides*, is widely distributed at the sandy bottoms of the East China Sea. However, the development and utilization of *Acaudina molpadioides* have not received extensive attention, resulting in very low prices for this species. In our previous paper, we had studied the antioxidant activities and protective effect against H_2_O_2_-induced injury of hydrolysates of collagen from *Acaudina molpadioides* by using alkaline protease [22]. However, there has been very little information available about the properties and functions of collagen from *Acaudina molpadioides*. The analysis of total collagen from *Acaudina molpadioides* could expand its application fields, such as directly used as biomedical materials, rather than just used in the form of protein hydrolysate. Therefore, we chose to characterize collagen isolated from *Acaudina molpadioides* in this study, by various techniques such as amino acid composition, Fourier Transform Infrared Spectroscopy (FTIR) spectrum, and zeta potential. In addition, its antioxidant activity and protective effect against H_2_O_2_-induced injury in RAW264.7 cells were also evaluated. This research may hopefully provide a reference for biomedical and cosmeceutical applications for collagen from *Acaudina molpadioides* in the future.

## 2. Results and Discussion

### 2.1. Ultraviolet (UV) Absorption Spectrum

**Figure 1:**
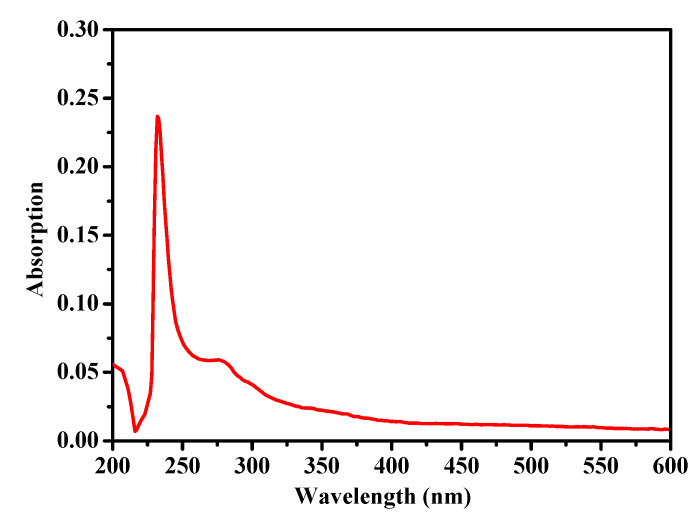
Ultraviolet (UV) spectra of collagen from the body wall of *A. molpadioides.*

It is well known that the UV absorption spectrum of a protein is determined by its amino acid composition. Although the maximum absorption peak of collagen was observed in the 210–240 nm range due to its triple helical structure, there were some differences found in its UV absorption spectrum compared to other sea cucumber collagens, due to its different amino acid composition. As shown in Figure 1, the collagen from the body wall of *A. molpadioides* exhibited maximum absorbance at 232 nm, suggesting that C=O, -COOH, and CONH_2_ moieties are present in the polypeptide chains of collagen. In addition, no obvious absorption peak was observed at 250–280 nm, indicating that levels of tryptophan, tyrosine, and phenylalanine were low in collagen from *A. molpadioides*. Compared to collagens from the sea cucumber *S. monotuberculatus* (218 nm) and *S. japonicus* (220 nm) [2,23], the maximum absorbance peak of collagen from *A. molpadioides* was more similar to collagen from large barbell catfish (233 nm) [24], showing that collagens from *A. molpadioides* and barbell catfish are likely similar in the amino acid composition.

### 2.2. Fourier Transform Infrared Spectroscopy (FTIR)

**Figure 2:**
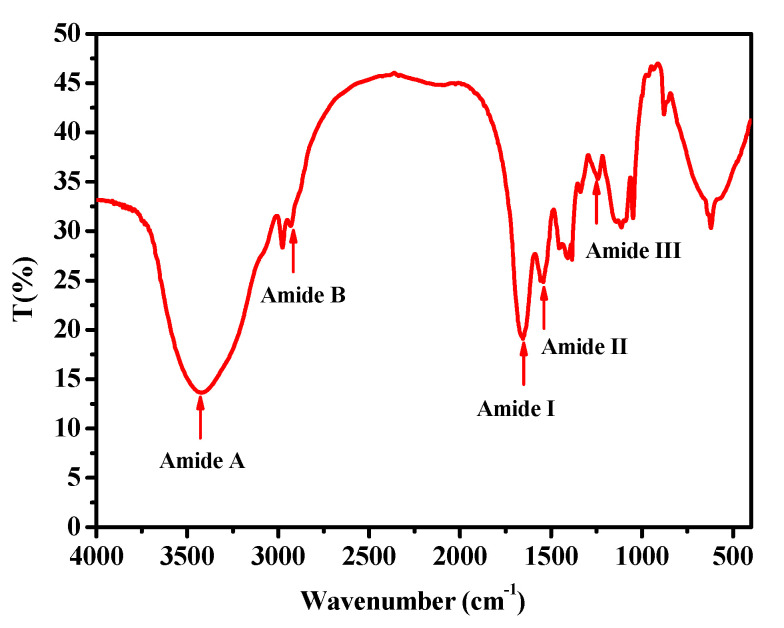
The fourier transform infrared spectroscopy (FTIR) spectrum of collagen from the body wall of *A. molpadioides.*

The FTIR spectrum (400–4000 cm^−1^) of collagen from the body wall of *A. molpadioides* is shown in Figure 2. Five major amide bands (amide A, amide B, amide I, amide II, and amide III) were found at wavenumbers of 3422.1, 2931.6, 1654.0, 1541.4, and 1241.9 cm^−1^. The amide A band is a free N-H stretching vibration, which is commonly located next to 3400–3440 cm^−1^. A lower wavenumber of amide A suggested more hydrogen bonding by N-H groups [25]. The amide A wavenumbers of acid-soluble collagen from the scales of miiuy croaker (*M. miiuy*) were in 3415 cm^−1^, indicating that the hydrogen-bonding numbers of collagen from *A. molpadioides* were less than that of collagen from *M. miiuy* [26]. The amide B band corresponded to an asymmetric stretch vibration of -NH_3_^+^ and =C-H, and an increase of free -NH_3_^+^ groups would result in a higher wavenumber [2,8,25]. The amide B band of collagen in this study was observed at 2931.6 cm^-1^, which was higher than that of collagen from *A. leucoprocta* (2926.4 cm^−1^). Amide I and amide II are considered the most important factors for evaluating the degree of molecular order and secondary structure of collagen [27]. When the molecular order is reduced, or the secondary structure changed, the wavenumber of the amide I band will be lower. The amide I band is related to the C=O stretching vibration, and its wavenumber is in the range of 1600–1700 cm^−1^. The wavenumber of the amide I band of collagen from the body wall of *A. molpadioides* was 1654.0 cm^−1^, indicating that the collagen retained its native secondary structure and molecular order. The amide II band is associated with N-H bending, and a lower wavenumber for this band indicates a higher structure order. The wavenumber of amide II of collagen was 1541.4 cm^−1^, suggesting a high structure order in collagen from *A. molpadioides*. The amide III band of collagen (1241.9 cm^−1^) was related to C-N stretching vibrations and N-H bending, demonstrating the existence of a helical structure.

### 2.3. Amino Acid Analysis

The amino acid composition of the collagen from the body wall of *A. molpadioides*, compared to collagen from *S. monotuberculatus* [2], *H. parva*, and calfskin [15] are presented in Table 1. The results show that the collagen from *A. molpadioides* is rich in glycine (Gly), alanine (Ala), glutamic acid (Glu), and proline (Pro), but poor in methionine (Met), tyrosine (Tyr), phenylalanine (Phe), lysine (Lys), and histidine (His). Gly represents about one-third of the total residues. Similar results were also observed in collagens from the sea cucumbers *S. monotuberculatus* and *H. parva*. In general, the content of Glu is closely related to the Isoelectric point (pI) of the protein. The pI value tends to decrease as the Glu content increases in collagen. The Glu content of collagen in this study was 100.8 residues/1000 residues, which was lower than that of Glu in collagen from the sea cucumber *S. monotuberculatus* (127 residues/1000 residues), but significantly higher than that of collagen from *H. parva* (74 residues/1000 residues) and calfskin (75 residues/1000 residues). This result suggested that the collagen from *H. parva* and calfskin probably had higher pI values than that from *A. molpadioides*. The imino acids hydroxyproline (Hyp) are unique amino acids in collagen and play important roles in stabilizing its triple helix structure. As shown in Table 1, the content of Hyp in collagen from *A. molpadioides* (57.5 residues/1000 residues) was similar to that in collagen from *H. parva* (62 residues/1000 residues) and *P. californicus* (58 residues/1000 residues) [13], but significantly lower than those of collagens from *S. monotuberculatus* (67 residues/1000 residues), *S. japonicus* (66 residues/1000 residues) [23], *M. miiuy* (89.5 residues/1000 residues), and calfskin (94 residues/1000 residues) [8]. This result suggests that the collagen from *A. molpadioides* may not be the best source of Hyp but it can still be used as a potential substitute for mammalian collagen due to the abundant resources and low price of *A. molpadioides*.

### 2.4. Zeta Potential

**Figure 3:**
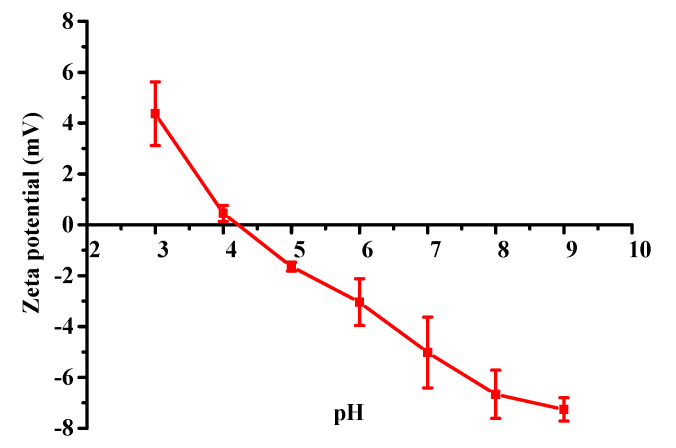
Zeta potential of collagen from *A. molpadioides* at different pH values.

Zeta potential is an important factor in the stability of collagen, which tends to form aggregates when the zeta potential of the collagen is close to zero [28]. Therefore, the zeta potential is also used to evaluate the isoelectric point (pI). The zeta potential of collagen from *A. molpadioides* was determined at pH values ranging from 3–9, and the result is shown in Figure 3. The data showed that the zeta potential values of collagen decreased as the pH was increased, being positively charged from pHs 3 to 4 and negatively charged from pHs 5 to 9. The pH was about 4.25 when the zeta potential value was zero, indicating that the pI of collagen from *A. molpadioides* was about 4.25. Generally, the pIs of collagens from different organisms are different due to differences in their amino acid compositions. It has been reported that the pI of collagen is always between pH 6.0 to 9.0 [29], such as the collagen from the tilapia skin (6.42) [30], the brown-banded bamboo shark (6.21) [31], the scales of the Miiuy Croaker (6.81) [26], and the seabass (6.46) [32]. The lower pI demonstrates that there are more acidic amino acids in collagen from *A. molpadioides*, especially glutamic acid, which was also proved by the amino acid analysis in Table 1. Low pI values due to high contents of glutamic acid were also observed in collagen from other sea cucumbers, including *S. monotuberculatus* [2], *A. leucoprocta* [18], *P. californicus* [13], and *S. japonicus* [33].

### 2.5. Antioxidant Activity

Many collagens from marine organisms have antioxidant, antibacterial, and anti-aging activities, which have attracted wide attention in the biomedical materials and cosmeceutical industries. The radical scavenging rate is an important indicator for evaluating antioxidant capability, which plays an important role in promoting wound healing and preventing skin aging. Therefore, scavenging assays of 2,2-diphenyl-1-picrylhydrazyl (DPPH) and 2,2’-azino-bis-3- ethylbenzothiazoline-6-sulfonic acid (ABTS) were implemented to evaluate the antioxidant activities of collagen from the body wall of *A. molpadioides*.

**Figure 4:**
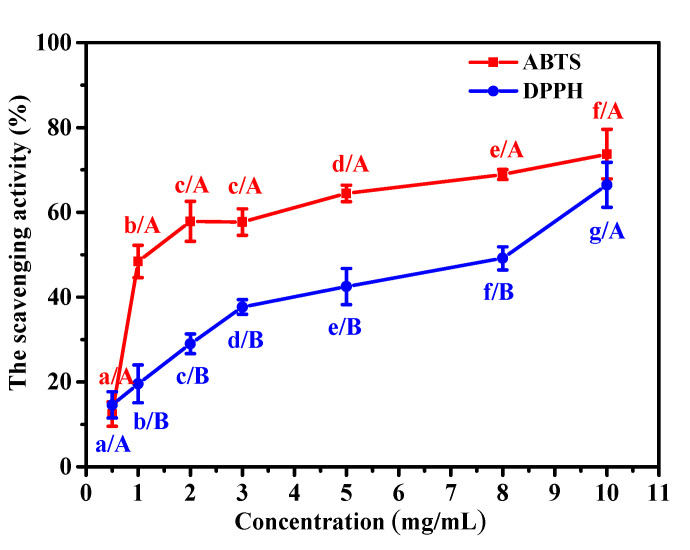
The scavenging activities of 2,2’-azino-bis-3-ethylbenzothiazoline-6-sulfonic acid (ABTS) and 2,2-diphenyl-1-picrylhydrazyl (DPPH) at different concentrations of collagen from *A. molpadioides*. (a–g) values with different letters indicated significant differences in the same samples at different concentrations (*p* < 0.05); (A–B) values with different letters indicated significant differences in the different samples at the same concentration (*p* < 0.05).

As shown in Figure 4, the collagen from *A. molpadioides* exhibited antioxidant activities against both DPPH and ABTS, and the higher the collagen content, the stronger the antioxidant activity. The scavenging activity of ABTS increased rapidly from 12.5% to 57.9% for collagen at concentrations ranging from 0.5 to 2 mg/mL (*p* < 0.05), but increased slowly to 73.7% at a concentration of 10 mg/mL (*p* < 0.05). For DPPH, the scavenging activity increased gradually from 14.6% to 66.5% at the tested concentrations of collagen (*p* < 0.05). The results indicated that collagen from the body wall of *A. molpadioides* revealed higher antioxidant activity for ABTS than DPPH, especially at low concentrations. Pal reported that the DPPH radical scavenging activity was about 20% for collagen from a carp swim bladder at a concentration of 1.0 mg/mL [34]. Similarly, acid-soluble collagen from the scales of the miiuy croaker has been shown to have 20% DPPH radical scavenging activity at a concentration 1.0 mg/mL [35]. The DPPH radical scavenging activity of collagen from *A. molpadioides* at 1.0 mg/mL in this study was 19.6%, which is similar to the results obtained with collagens from the carp swim bladder and the scales of the miiuy croaker. In addition, the ABTS radical scavenging activity for collagen from *A. molpadioides* at 1.0 mg/mL was 48.4%, which was significantly higher than that of collagen from the swim bladders of miiuy croakers (about 10%) at the same concentration [8]. It was reported that the antioxidant activity of collagen may be related to its ability to reduce hydroperoxide content, inactivate active oxygen and scavenge free radicals [36].

### 2.6. Protective Effect against H_2_O_2_-induced Injury on RAW264.7 Macrophage Cells

**Figure 5:**
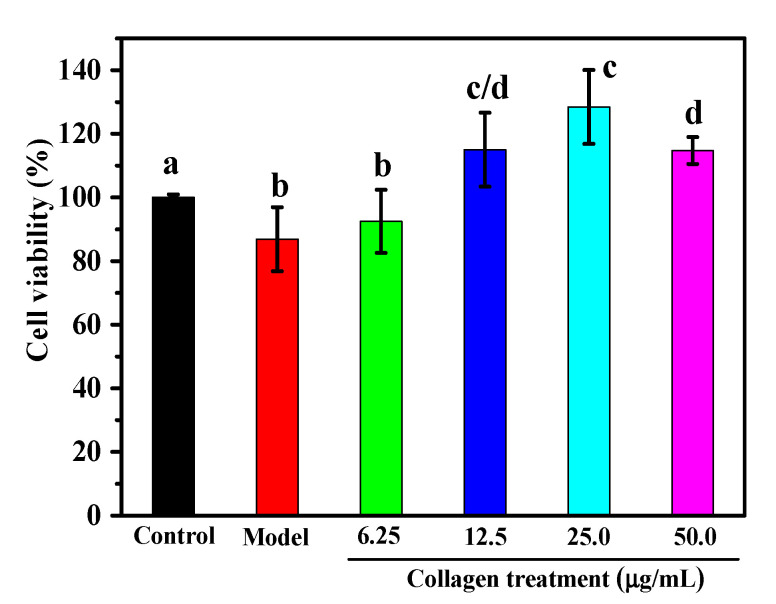
Effect of collagen from *A. molpadioides* on H_2_O_2_-induced injury RAW264.7 cells viability. The cell viability was measured after incubation for 24 h in medium with or without collagen and exposed to H_2_O_2_ (1 mM) for 4 h. Values with different letters were significantly different (*p* < 0.05).

H_2_O_2_ is a Reactive Oxygen Species (ROS) that may damage or even kill cells through direct oxidation of biomolecules (lipids, proteins, DNA) or by triggering intracellular pathways [35]. Macrophages are essential for identifying and eliminating microbial pathogens in the host defense system and are the main targets of pro-oxidants. It is commonly used in macrophages to study apoptosis or oxidative stress-mediated cell damage [35,37,38]. As shown in Figure 5, the viability of RAW264.7 cells incubated with 1 mM H_2_O_2_ for 4 h was 86.8% of the control value. In order to evaluate the protective effect of collagen from *A. molpadioides* against H_2_O_2_-induced injury on RAW264.7 macrophage cells, 6.25, 12.5, 25, and 50 μg/mL of collagen were selected to study the effects of collagen on cell viability and morphology. As the concentration of collagen increased from 0 to 25 μg/mL, as shown in Figure 5, the cell viability increased from 86.8% to 128.5%. However, a small decline in cell viability was observed at a concentration of 50 μg/mL, indicating that high concentrations of collagen may be toxic to RAW264.7 cells. When the concentration of collagen was in the range of 12.5–50 μg/mL, the cell viability was significantly improved compared to the control and H_2_O_2_ model groups.

**Figure 6:**
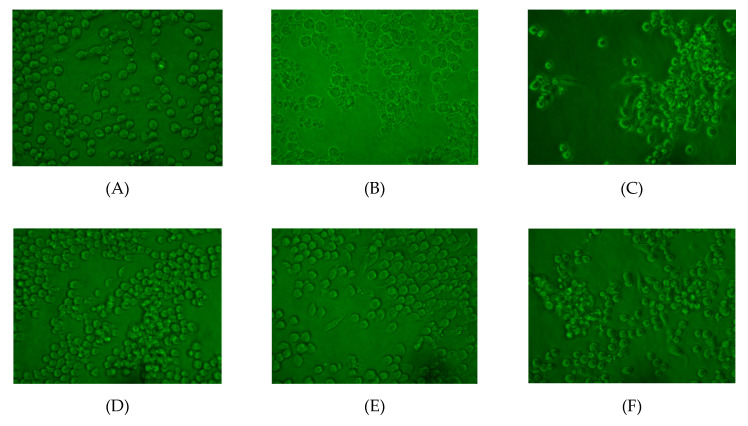
Effect of collagen from *A. molpadioides* on H_2_O_2_-induced injury RAW264.7 cell morphology. The cells of each group were directly observed under an inverted microscope, and the background of the pictures were set to green. (**A**) control group; (**B**) model group (1 mM H_2_O_2_); (**C**) 6.25 µg/mL collagen + 1 mM H_2_O_2_; (**D**) 12.5 µg/mL collagen + 1 mM H_2_O_2_; (**E**) 25.0 µg/mL collagen + 1 mM H_2_O_2_; (**F**) 50.0 µg/mL collagen + 1 mM H_2_O_2_.

As shown in Figure 6, the morphologies of RAW264.7 cells in the control group were very regular and intact, while cells in the model group were largely damaged. When pretreated with 6.25 μg/mL of collagen (C group), the cell morphology was significantly improved, but some broken cells were still visible. As the concentration of collagen increased to 12.5 or 25.0 μg/mL, the cell morphology was substantially improved, and no damaged cells were observed. However, a small number of damaged cells were observed in group F (50 μg/mL of collagen), which was probably due to the high concentration of collagen poisoning these cells.

**Figure 7:**
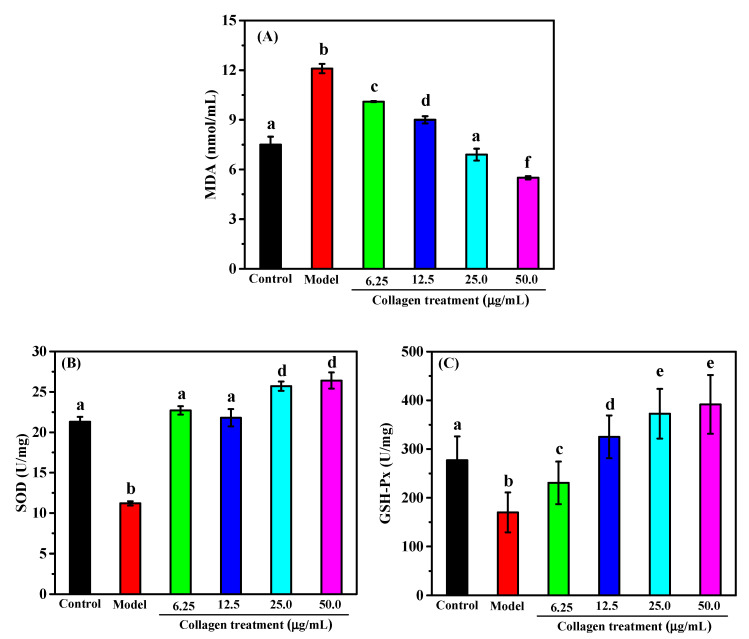
Effect of collagen from *A. molpadioides* on levels of Malondialdehyde (MDA) (**A**), Superoxide Dismutase (SOD) (**B**), and Glutathione Peroxidase (GSH-Px) (**C**) in RAW264.7 cells induced by H_2_O_2_. Values with different letters were significantly different (*p* < 0.05).

Malondialdehyde (MDA) is the major product of the reaction of free radicals with lipids in the body. The level of MDA indirectly reflects the level of severity of the cellular attack by free radicals [39]. As shown in Figure 7A, the MDA level of the H_2_O_2_-induced model group was significantly higher than that of the control group, indicating that the cells were severely damaged by H_2_O_2_ stimulation. The collagen treatment group showed a significantly lower MDA content compared to the H_2_O_2_-induced model cells in a dose-effect relationship. When the concentration of collagen reached 25 μg/mL, the level of intracellular MDA was comparable to that of the control group. When the concentration of collagen continued to increase to 50 μg/mL, the level of MDA was even lower than that of the control group. This result indicates that collagen could effectively alleviate the oxidative stress damage of free radicals to the body.

Both Superoxide Dismutase (SOD) and Glutathione Peroxidase (GSH-Px) are important components of antioxidant enzymes in cells, playing important roles in the balance between antioxidants and oxidative stress [40]. SOD catalyzes the disproportionation of peroxy anions for the scavenging of free radicals. GSH-Px catalyzes the reaction of GSH with peroxide, removing toxic and harmful hydrogen peroxide. Therefore, the levels of antioxidant enzyme activities in the body directly reflect the level of dynamic balance regulation of cells under oxidative stress [41]. It could be seen from Figure 7B that the SOD activity of the model group was significantly decreased compared to the control group, and that collagen pretreatment could effectively increase SOD activity in the model cells. The activities of SOD in the 6.25 μg/mL and 12.5 μg/mL of collagen pretreatment groups had increased to levels that were no longer significantly different from that of the control group. When the concentration of collagen increased to 25 μg/mL or 50 μg/mL, the SOD activities in the model cells were even higher than in normal cells (control group). Figure 7C shows that the GSH-Px activity in the model group was significantly lower than that of the control group but increased significantly after pretreatment with collagen. When the collagen concentration was ≥12.5 μg/mL, the GSH-Px activities in the H_2_O_2_-induced model cells increased to levels even higher than those of the normal cells (control group).

In summary, the collagen from *A. molpadioides* is able to significantly improve the cell viability and morphology of H_2_O_2_-induced RAW264.7 cells. The protective effect of collagen against H_2_O_2_-induced injury in RAW264.7 cells may be related to the fact that it significantly reduced the intracellular MDA content and increased the enzymatic activities of both SOD and GSH-Px. This study suggested that collagen from *A. molpadioides* may be a promising source for the development of natural antioxidants, and could be effectively used in the beauty and cosmetics industry.

## 3. Materials and Methods

### 3.1. Materials

Sea cucumber *A. molpadioides* was obtained from the wharf of Xiangshan, in the Zhejiang Province of China. The protein marker was purchased from Sigma-Aldrich (Shanghai, China). The 2,2-dipehnyl-1-picryldydrazyl (DPPH) and dimethyl sulfoxide (DMSO) were purchased from Sigma-Aldrich (Shanghai, China). The malondialdehyde (MDA), glutathione peroxidase (GSH-Px), and superoxidase dismutase (SOD) assay kits were provided by the Nanjing Jiancheng Bioengineering Institute (Nanjing, China). All other chemicals used were of analytical grade.

### 3.2. Extraction of Collagen from A. Molpadioides

The collagen was extracted from the body wall of *A. molpadioides* according to previously reported methods [18]. The *A. molpadioides* was washed and cut into small pieces. The samples were soaked in 10 times the volume of ethylenediamine tetraacetic acid (EDTA, 0.2 M, pH 8.0) and NaOH (0.1 M) in turn. After 48 h, the precipitate after centrifugation was transferred to a 10-fold volume of acetic acid (0.5 M) containing 0.1% (*w/v*) pepsin at 4 °C for 48 h. NaCl was added into the filtrate obtained by two layers of cotton cloth with the final concentration of 0.8 M. The precipitate was collected by centrifugation, then dialyzed, and freeze-dried to obtain collagen.

### 3.3. UV Absorption Spectrum

The UV absorption spectra of collagen were performed using a spectrophotometer (UV-1800, Mapada Instruments Co., Ltd., Shanghai, China) from 200 to 600 nm. Collagen was dissolved in 0.5 M acetic acid solution with a final concentration 0.1 mg/mL.

### 3.4. FTIR Spectrum Analysis

The FTIR spectrum of collagen was performed by the spectrophotometer Nicolet 6700 (Thermo Fisher Scientific Inc., Waltham, MA, USA). The mixture with 0.1 mg collagen and 10 mg KBr was pressed into a disk for spectrum recording. The infrared spectroscopy (IR) spectra were recorded in the range of 4000–400 cm^−1^ at a rate of 2 cm^−1^ per point. The final spectrum curve was obtained by Excel 2007.

### 3.5. Amino Acid Analysis

The amino acid composition of collagen was analyzed using a previously described protocol [42]. The collagen samples were hydrolyzed with 6 M HCl at 110 °C for 24 h. The hydrolysates were then used to analyze the amino acid composition on an automated HITACHI L8900 amino acid analyzer (Hitachi High-Technologies Corporation, Tokyo, Japan).

### 3.6. Zeta Potential

Collagen was dissolved in 0.2 M acetic acid to a final concentration of 0.1 mg/mL. The zeta potential of collagen was measured using a Malvern Zetasizer Nano ZS90 (Malvern Instruments Ltd., UK). The tested pH (3–9) was adjusted with 1 M NaOH and 1 M HCl.

### 3.7. Antioxidant Activity

#### 3.7.1. DPPH Radical Scavenging Activity

DPPH was dissolved in absolute ethanol with final concentration 0.04 mol/mL. Two mL DPPH solution, and 1 mL ethanol were added into 1 mL of the sample solution. The mixture was incubated for 30 min at room temperature. After centrifugation at 5000 rpm for 5 min, the absorbance of the supernatant was measured at 517 nm. A control (containing 2 mL DPPH solution, 1 mL water, and 1 mL ethanol) and a blank (containing 1 mL sample solution and 3 mL ethanol) were prepared. The antioxidant activity of the sample was evaluated by the scavenging rate of DPPH with the Equation (1):

DPPH scavenging rate (%) = (A_0_ − A + A_1_)/A_0_ × 100% (1)

where A was the sample absorbance; A_0_ was the control absorbance; A_1_ was the blank absorbance.

#### 3.7.2. ABTS Radical Scavenging Activity

Four hundred and forty μL of 140 mM potassium persulfate solution and 25 mL of 7 mM ABTS solution were mixed and incubated for 14 h. The mixture was diluted with methanol to an absorbance of 0.7 ± 0.002 as a working solution. 0.15 mL of the sample solution was added into 2.85 mL of the working solution. After being incubated for 10 min, the absorbance was measured at 734 nm. The antioxidant activity of the sample was evaluated by the scavenging rate of ABTS with the Equation (2):

ABTS scavenging rate (%) = (A_c_ − A_s_)/A_c_ × 100% (2)

where A_c_ was the absorbance without the sample and A_s_ was the absorbance with the sample.

### 3.8. Cell Morphology Observation and Measurement of Cell Viability

RAW264.7 cells were seeded in 96-well plates at a density of 1 × 10^5^ /mL in culture medium and incubated for 24 h at 37 °C and 5% CO_2_. The control group and the model group were supplemented with 200 µL medium, and the experimental groups each received 200 µL of a solution with a different concentration of collagen (6.25, 12.5, 25, and 50 µg/mL). After incubation for 24 h at 37 °C and 5% CO_2_, the model group and the experimental groups were then exposed to H_2_O_2_ (1 mM) for 4 h. The morphology of each group was observed under an inverted microscope. Two hundred μL 3-(4,5-dimethylthiazol-2-yl)-2,5-diphenyltetrazolium bromide (MTT) was added to each well, followed by 4 h of incubation at 37 °C. One hundred and fifty μL DMSO was added into each well and incubated for 20 min. The absorbance of the plate was determined using a microplate reader at 490 nm. The cell viability was calculated according to the following formula:

cell viability (%) = 100 × (absorbance of treatment/absorbance of control)

### 3.9. Assays for the Levels of MDA, SOD, and GSH-Px

Two mL of RAW264.7 cells with a cell concentration of 1.0 × 10^5^ /mL were inoculated into six-well plates and cultured for 24 h at 37 °C and 5% CO_2_. The cells were grouped and cultured as described in Section 3.8. Next, the cells were washed twice with phosphate buffered solution (PBS) and lysed with cell lysates. After centrifugation at 1000 rpm for 10 min at 4 °C, the supernatant fractions were collected. Assays for quantification of the levels of MDA, SOD, and GSH-Px in the collagen were conducted according to the kit instructions, using spectrophotometric methods.

### 3.10. Statistical Analysis

All experiments were carried out in triplicate. Significant differences between means were measured by Duncan’s multiple range test (*p* < 0.05). The analysis was performed using the software SPSS 19.0 (SPSS Inc., Chicago, IL, USA).

## 4. Conclusions

In this study, the physicochemical properties, antioxidant activity, and protective effect against H_2_O_2_-induced injury in RAW264.7 cells of collagen from *A. molpadioides* were investigated. The collagen from *Acaudina molpadioides* has a lower isoelectric point compared to collagens from tilapia and th miiuy croaker, due to its higher content of Glu. It showed scavenging activities for both DPPH and ABTS radicals in a dose-dependent manner. Furthermore, the collagen exhibited protective effects against H_2_O_2_-induced injury in RAW264.7 cells by reducing their MDA contents and increasing the activities of SOD and GSH-Px. This study will hopefully provide helpful information for the further application of collagen from *A. molpadioides* in the beauty and biomedical industries.

## Figures and Tables

**Table 1:** Amino acid composition of collagen in comparison with the amino acid composition of collagens from other sea cucumbers (residues/1000 residues).

Amino Acid	*A. Molpadioides*	*S. Monotuberculatus*	*H. Parva*	Calf Skin
Aspartic acid (Asp)	63.7	70	50	45
Threonine (Thr)	40.8	34	ND ^1^	ND ^1^
Serine (Ser)	40.7	26	20	39
Glutamic acid (Glu)	100.8	127	74	75
Glycine (Gly)	336.9	320	270	330
Alanine (Ala)	127.9	92	91	119
Valine (Val)	21.1	21	18	21
Methionine (Met)	5.7	6	5	6
Isoleucine (Ile)	7.1	15	4	11
Leucine (Leu)	19.6	20	16	23
Tyrosine (Tyr)	10.5	6	4	3
Phenylalanine (Phe)	8.0	10	6	3
Lysine (Lys)	4.1	6	7	26
Histidine (His)	4.6	9	ND ^1^	5
Arginine (Arg)	45.1	71	49	50
Proline (Pro)	98.5	84	96	121
Hydroxyproline (Hyp)	57.5	67	62	94
Imino Acid	156	151	158	215

^1^ not detected.
